# Prevalence and Determinants of Sexual Dysfunction in Male Diabetic Patients in Saudi Arabia: A Cross-Sectional Study

**DOI:** 10.7759/cureus.82901

**Published:** 2025-04-24

**Authors:** Sherif M Zaki, Mohammed A Zahra, Saleem Alshobaki, Yousef Salamah, Nawaf Al-Nazzawi, Hussain Al-Taffi, Abdulaziz Yassin, Obai Yamani

**Affiliations:** 1 Physiological Sciences Department, MBBS Program, Fakeeh College for Medical Sciences, Jeddah, SAU; 2 Medical Student, MBBS Program, Fakeeh College for Medical Sciences, Jeddah, SAU

**Keywords:** a cross-sectional study, associated risk factors, diabetic patients, male sexual dysfunction, saudi arabia

## Abstract

Introduction

In Saudi Arabia, sexual health among diabetic men is one aspect that is less explored, unlike other complications related to diabetes. This study aimed to look at the prevalence of erectile dysfunction (ED) and premature ejaculation (PE) in male Saudi patients with diabetes and their risk factors.

Methods

A cross-sectional study targeting 100 male diabetic patients was carried out at Dr. Soliman Fakeeh Hospital in Jeddah, Kingdom of Saudi Arabia. Participants provided demographic and medical history information and underwent laboratory investigations, including glycated hemoglobin (HbA1c), lipid profiles, and markers of renal function. We evaluated sexual dysfunction using the International Index of Erectile Function and the Premature Ejaculation Diagnostic Tool.

Results

Eighty-five percent of participants had ED and 47% had PE. Erectile dysfunction (ED) was significantly associated with age (OR 1.51, p < 0.0), marital status (OR 1.48, p < 0.05), smoking (OR 2.90, p < 0.05), and diabetes duration (OR 2.90, p < 0.05). For PE, the factors marital status, smoking, and diabetes duration were found to be significant with ORs of 0.63, 1.81, and 1.31, respectively (p < 0.05). The diabetes patients with higher levels of triglycerides, hypercholesterolaemia, and abnormal levels of HDL and LDL had a higher risk for both conditions.

Conclusion

The increased incidences of ED and PE emphasise the importance of a proper diagnostic approach. An important step would be to implement strategies to quit smoking, control blood sugar levels, manage lifestyle habits, and regulate lipids. Screening for sexual dysfunctions and assessing lipid profiles should be part of diabetes management in order to enhance the patient's life.

## Introduction

Diabetes mellitus is a chronic condition with profound systemic implications. Among the complications that affect diabetic patients is sexual dysfunction. Male sexual dysfunction (SD) includes erectile dysfunction (ED), ejaculatory dysfunction, orgasmic dysfunction, and hypoactive sexual desire disorder (HSDD) [1]. ED has the highest morbidity, followed by premature ejaculation (PE) [2]. Disorders such as these are a major cause of deterioration in quality of life and personal relationships and can further exacerbate the psychological and physical load faced by sufferer groups [3]. These conditions are often unrecognised and untreated, though they severely impact both quality of life and psychological well-being [4].

Endothelial dysfunction, which includes lower bioavailability of nitric oxide and poor vasodilation, is a major cause of ED in diabetics. These processes are essential for achieving an erection. Additionally, diabetic neuropathy contributes to sensory deficits and autonomic dysfunction, compounding the difficulty in achieving or maintaining sexual performance [5]. 

Premature ejaculation (PE) is indeed associated with diabetes, although the relationship is complex and multifaceted. PE is common among many diabetic patients due to autonomic neuropathy, which leads to an overactive sympathetic nervous system [6]. Furthermore, stress and anxiety associated with diabetes elevate the threshold for ejaculation further through more stimulation of the sympathetic system [7]. Diabetes is also known to alter the hormones and thus the normal function during sexual activity. In the end, there is a much higher incidence of PE in diabetic patients who already suffer from erectile dysfunction [8].

The presence of diabetes increases the risk of sexual dysfunction threefold when compared to non-diabetic populations [9]. The prevalence of ED alone in diabetic men ranges from 35% to 75% [10]. Large population-based studies have recognised diabetes as the most important chronic disease associated with ED [11]. According to a meta-analysis of worldwide data from 145 studies representing 88,577 men, the overall estimated prevalence of ED was 52.5% [12]. This was much higher in men with diabetes than in healthy men, with an odds ratio of 3.62 (95% CI, 2.53 to 5.16). Studies using the International Index of Erectile Function-5 (IIEF-5) scale reported a higher prevalence of 82.2% [13].

Male sexual dysfunction also has far-reaching psychosocial implications, negatively impacting marital relationships, self-esteem, and overall quality of life [14]. Despite these severe consequences, many diabetic men do not seek help due to cultural shames or a lack of awareness, particularly in Middle Eastern societies, where discussing sexual health remains a sensitive topic [15]. With a high prevalence of diabetes, Saudi Arabia is giving us an opportunity to study this issue in a unique context. Saudi Arabia has extensively studied the complications of diabetes, such as nephropathy, neuropathy, and retinopathy, but the sexual health of diabetic men remains largely unexplored. The current study aims to fill this gap by assessing the prevalence of ED and PE among diabetic patients in Saudi society and the associated risk factors. Focusing on a Saudi cohort, this research will reveal the specific epidemiological features of sexual dysfunctions in this population and contribute to the design of culturally appropriate and clinically effective management strategies.

## Materials and methods

A total of 100 male diabetic patients who visited Dr. Soliman Fakeeh Hospital, Jeddah, Saudi Arabia, between August and December 2024 were recruited for this cross-sectional, non-interventional, observational study. All participants gave informed consent prior to participation. We collected and stored the data in an encrypted file to safeguard the volunteers' identity and privacy. This study received ethical approval in accordance with the principles stated in the Declaration of Helsinki from the Institutional Review Board of Fakeeh College for Medical Sciences (approval number: 506/IRB/2023).

The study included participants who met the following criteria: a confirmed diagnosis of diabetes, an age range between 18 and 60 years, and the provision of written informed consent. We excluded individuals who were not sexually active, had severe conditions such as mental illnesses or physical disabilities that hindered their ability to participate in sexual health assessments, or lacked complete survey data.

Socio-demographic information, including age (years), height (cm), weight (kg), body mass index (BMI) (kg/m2), smoking history, and marital status, was collected through direct interviews. Additionally, medical records provided data on blood pressure, diabetes as the primary diagnosis, and associated comorbidities.

We got glycated hemoglobin (HbA1c), fasting plasma glucose (mg/dL), haemoglobin level (g/dL), total cholesterol (mmol/L), triglyceride (mmol/L), low-density lipoprotein cholesterol (LDL) (mmol/L), high-density lipoprotein cholesterol (HDL) (mmol/L), serum creatinine (µmol/L), estimated glomerular filtration rate (eGFR) (mL/min/1.73 m²), urea nitrogen (BuN) (mmol/L), urinary albumin-to-creatinine ratios (U.ACR) (mg/L), and blood uric acid (BUA) (µmol/L).

We evaluated the subjects' sexual dysfunction using a five-item version of the International Index of Erectile Function (IIEF-5) and the Premature Ejaculation Diagnostic Tool (PEDT). IIEF-5 includes five items that address erectile function and satisfaction with sexual intercourse. Additional analysis of the questionnaire helps to distinguish the presence and severity levels of ED: normal ED (22-25 points), mild ED (12-21 points), moderate ED (8-11 points), and severe ED (1-7 points) [16]. We used PEDT to assess PE. PEDT scores ≥11 indicate PE diagnosis; 9 or 10 refer to probable PE, while ≤ 8 indicates the nonexistence of PE [17]. Simultaneously, we considered PE if a patient's IELT (intravaginal ejaculatory latency time) was less than 3 minutes and they experienced poor sexual satisfaction.

The data were statistically analysed using IBM SPSS Statistics version 26 (IBM Corp., Armonk, NY, USA). We checked the normality of the data at the first stage using the Shapiro-Wilk test. We calculated descriptive statistics to characterise the participants, which included means (M) and standard deviations (SD) for continuous variables and frequencies with percentages for categorical variables. We used relative risks (RR) and odds ratios (OR) with 95% confidence intervals to analyse the association of risk factors for the development of ED and PE. For all tests, a p-value <0.05 was considered statistically significant.

## Results

Analysis of findings on premature ejaculation

The prevalence pattern of PE varies among different subgroups within patients with diabetes. PE patients (43±10 years) were comparable in age to patients without PE (44±10 years) and borderline PE (39±11 years). No significant difference in height, weight, and BMI was observed between groups, unlike blood pressure parameters, which were all significantly different between groups, with borderline PE (BP 89±9 mmHg) being higher than the other groups in diastolic BP. There were no significant group differences in HbA1c, fasting plasma glucose, or lipid profiles (cholesterol, triglycerides (TG), or low-density lipoprotein (LDL)). Renal function markers (serum creatinine, eGFR, and urinary albumin to creatinine ratio) were stable between the groups. On the other hand, borderline PE patients showed slightly lower haemoglobin levels (11.3 ± 3 g/dL) (Table 1).

**Table 1:** Premature ejaculation and associated factors in diabetic patients (Mean ± SD) *Statistically significant using t-test, HBA1c (haemoglobin A1c), Hb (haemoglobin), U.ACR (urinary albumin-to-creatinine ratio), TG (triglycerides), LDL (low-density lipoprotein), HDL (high-density lipoprotein), eGFR (estimated glomerular filtration rate), and BUN (blood urea nitrogen).

	Premature ejaculation (PE)				p-value
	No PE	Borderline PE	PE	Total	
Age (years)	44±10	39±11	43±10	43±11	0.438
Height (cm)	158±13	156±8	161±10	159±11	0.357
Weight (Kg)	77±17	81±10	77±19	77±18	0.845
BMI (kg/m2)	31±8	33±6	30±8	31±8	0.500
Systolic blood pressure (mmHg)	125±9	123±9	124±11	124±10	0.900
Diastolic blood pressure (mmHg)	81±14	89±9	78±12	80±13	0.01*
HBA1c	6±1	6±1	6±1	6±1	0.562
Fasting plasma glucose (mg/dL)	96±15	106±15	98±16	98±15	0.235
Hb level (g/dL)	12±2	11±3	12±2	12±2	0.447
Total cholesterol (mmol/L)	5.7±2.1	6.2±2.3	5.8±2	5.8±2.1	0.852
TG (mmol/L)	4.3±2	5.1±1.8	4.4±1.9	4.4±1.9	0.615
LDL (mmol/L)	3.3±2	3.6±1.7	3.4±2.2	3.4±2.1	0.922
HDL (mmol/L)	1.33±.8	1.46±1.01	1.45±.86	1.39±.84	0.767
Serum creatinine (μmol/L)	80±25	77±29	82±22	81±23	0.873
eGFR (mL/min/1.73 m^2^)	92±19	91±16	92±18	92±18	0.974
U.ACR (mg/L)	63±36	65±38	61±36	62±36	0.921
BUN (mmol/L)	6±2	6±3	7±3	6±3	0.510
Blood uric acid (μmol/L)	264±67	285±58	272±71	269±68	0.687

**Figure 1:**
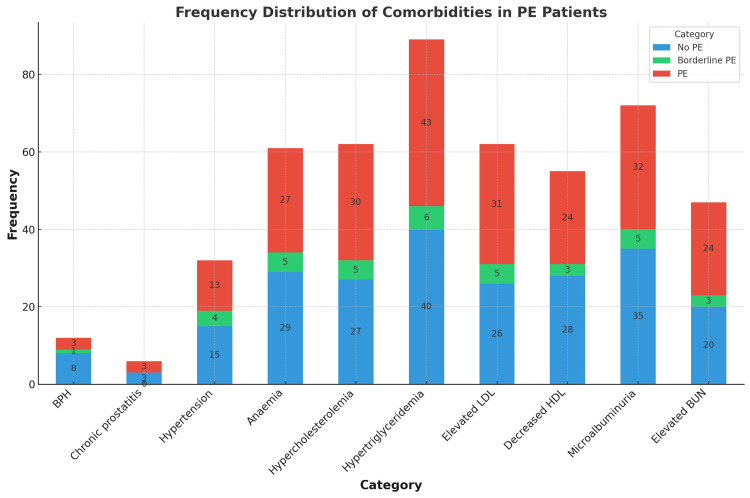
Frequency distributions of comorbidities in PE patients PE (premature ejaculation), LDL (low-density lipoprotein), HDL (high-density lipoprotein), and BUN (blood urea nitrogen).

The two age groups, 20-44 years and 45-64 years, had equal distributions of PE. Married status at 40% accounted for most PE cases. Obesity was the most frequent response to BMI categories in PE patients, at 20%; overweight was second at 11%. A minority of PE sufferers were current smokers (9%), while 7% were ex-smokers. Major comorbid conditions are erectile dysfunction (39%); hypertriglyceridemia (43%); hypercholesterolaemia (30%); elevated LDL (31%); and microalbuminuria (32%) (Table 2, Figure 1).

**Table 2:** Prevalence of demographics and lifestyle in PE (frequency and percentage of total) *Statistically significant by using Chi-Square test, PE (premature ejaculation), BPH (benign prostatatic hyperplasia), TG (triglycerides), LDL (low-density lipoprotein), HDL (high-density lipoprotein), eGFR (estimated glomerular filtration rate), and BUN (blood urea nitrogen).

		Premature ejaculation (PE)			Total	p-value
		No PE	Borderline PE	PE		
Age group	20-44 years	21 (21%)	4 (4%)	25 (25%)	50 (50%)	0.432
	45-64 years	25 (25%)	3 (3%)	22 (22%)	50 (50%)	
Marital status	Married	36 (36%)	6 (6%)	40 (40%)	82 (82%)	0.668
Smoking status	Non-smoker	33 (33%)	5 (5%)	36 (36%)	74 (74%)	0.652
	Smoker	13 (13%)	2 (2%)	11 (11%)	26 (26%)	
BMI category	Underweight	1 (1%)	0 (0%)	1 (1%)	2 (2%)	0.472
	Normal weight	9 (9%)	0 (0%)	15 (15%)	24 (24%)	
	Overweight	10 (10%)	3 (3%)	11 (11%)	24 (24%)	
	Obese	26 (26%)	4 (4%)	20 (20%)	50 (50%)	
Type of diabetes	Type 1 diabetes	9 (9%)	1 (1%)	6 (6%)	16 (16%)	0.665
	Type 2 diabetes	37 (37%)	6 (6%)	41 (41%)	84 (84%)	
Duration of diabetic illness	Less than 10 years	18 (18%)	0 (0%)	9 (9%)	27 (27%)	0.024*
	More than 10 years	28 (28%)	7 (7%)	38	73 (73%)	
Comorbidity	BPH	8 (8%)	1 (1%)	4 (4%)	13 (13%)	0.256
	Chronic prostatitis	4 (4%)	0 (0%)	5 (5%)	9 (9%)	0.786
	Hypertension (DBP≥ 90 mmHg)	15 (15%)	4 (4%)	13 (13%)	32 (32%)	0.294
	Anaemia (Hb < 13 g/dL)	29 (29%)	5 (5%)	27 (27%)	61 (61%)	0.772
	Hypercholesterolemia (˃ 5.2 mmol/L)	27 (27%)	5 (5%)	30 (30%)	62 (62%)	0.762
	Hypertriglyceridemia (TG˃ 1.7 mmol/L)	40 (40%)	6 (6%)	43 (43%)	89 (89%)	0.752
	Elevated LDL (≥2.6 mmol/L)	26 (26%)	5 (5%)	31 (31%)	62 (62%)	0.559
	Decreased HDL (<1.6 mmol/L)	28 (28%)	3 (3%)	24 (24%)	55 (55%	0.509
	Microalbuminuria	35 (35%)	5 (5%)	32 (32%)	72 (72%)	0.691
	Elevated BUN	20 (20%)	3 (3%)	24 (24%)	47 (47%)	0.745
Creatinine (65-115 µmol/L)	Normal	26 (26%)	3 (3%)	29	58	0.909
	Elevated	5 (5%)	1 (1%)	5 (5%)	11 (11%)	
	Decreased	15 (15%)	3 (3%)	13 (13%)	31 (31%)	
Erectile dysfunction	No erectile dysfunction	6 (6%)	1 (1%)	8 (8%)	15 (15%)	0.383
	Mild erectile dysfunction	25 (25%)	3 (3%)	21 (21%)	49 (49%)	
	Moderate erectile dysfunction	10 (10%)	0 (0%	8 (8%)	18 (18%)	
	Severe erectile dysfunction	5 (5%)	3 (3%)	10 (10%)	18 (18%)	
eGFR	G1 (normal, no CKD)	22 (22%)	4 (4%)	26 (26%)	52 (52%)	0.740
	G2 (No CKD)	24 (24%)	3 (3%)	21 (21%)	48 (48%)	
Total		46 (46%)	7 (7%)	47 (47%)	100 (100%)	

**Figure 2:**
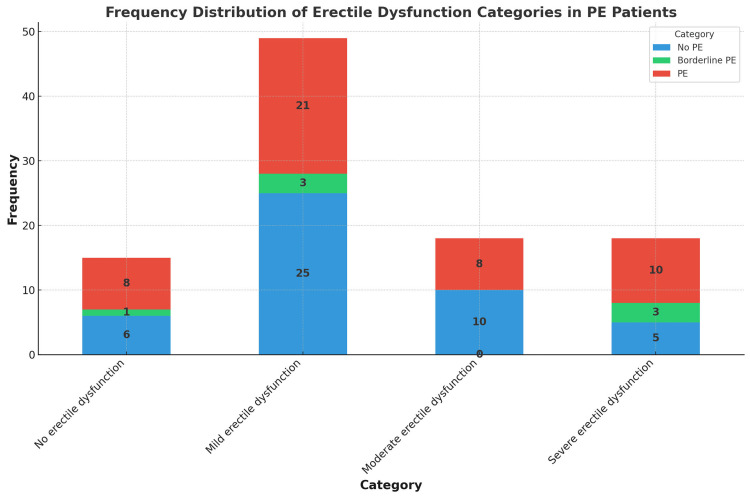
Frequency distribution of erectile dysfunction categories in PE patients PE (premature ejaculation)

Seven percent of the cases were classified as borderline PE, and 47% had PE. Erectile dysfunction was noted in 85% of patients, of whom 49% had mild ED, 18% moderate ED, and 18% severe ED. ED and PE were not present in 15% and 46% of the cases, respectively. ED and PE occurred simultaneously in 39% of the cases. Figure 2 illustrates the frequency of coexistence between ED and PE, showcasing the distribution of PE statuses across different levels of ED severity.

**Figure 3:**
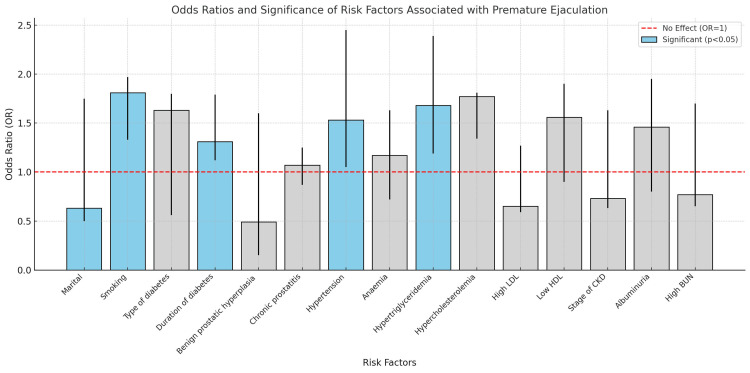
Odds ratio for risk factors associated with premature ejaculation OD (odds ratio), RR (relative risk), CI (confidence interval), LDL (low-density lipoprotein), HDL (high-density lipoprotein), CKD (chronic kidney disease), and BUN (blood urea nitrogen).

Looking at odds ratios (ORs) and relative risks (RRs) for things that are linked to PE gives us important information about how these things are connected to the condition. There were statistically significant links (p < 0.05) between smoking (OR: 1.81, 95% CI: 1.33-1.97), high blood pressure (OR: 1.53, 95% CI: 1.05-2.45), and high triglycerides (OR: 1.68, 95% CI: 1.19-2.39), which suggests that these conditions may make getting PE more likely. The risk of PE was significantly associated with duration of diabetes (OR: 1.31, 95% CI: 1.12-1.79). But benign prostatic hyperplasia (BPH), low HDL, and chronic prostatitis did not have significant associations (p ≥ 0.05), which means they were not linked to PE in the people who were studied (Table 3, Figure 3).

**Table 3:** Odds ratio for risk factors associated with premature ejaculation *Statistically significant by using logistic regression, OD (odds ratio), RR (relative risk), CI (confidence interval), LDL (low-density lipoprotein), HDL (high-density lipoprotein), CKD (chronic kidney disease), and BUN (blood urea nitrogen).

Risk Factor	RR	OR	95% CI Lower	95% CI Upper	p-value
Marital status	0.79	0.63	0.5	1.75	0.04*
Smoking	1.89	1.81	1.33	1.97	0.04*
Type of diabetes	1.28	1.63	0.56	1.8	0.42
Duration of diabetes	1.58	1.31	1.12	1.79	0.01
Benign prostatic hyperplasia	0.71	0.49	0.15	1.6	0.25
Chronic prostatitis	1.04	1.07	0.87	1.25	1
Hypertension	1.28	1.53	1.05	2.45	0.01*
Anaemia	1.09	1.17	0.72	1.63	0.84
Hypertriglyceridemia	1.82	1.68	1.19	2.39	0.03*
Hypercholesterolemia	1.87	1.77	1.34	1.73	0.54
High LDL	0.8	0.65	0.59	1.27	0.31
Low HDL	1.27	1.56	0.9	1.9	0.32
Stage of CKD	0.85	0.73	0.63	1.63	0.54
Albuminuria	1.24	1.46	0.8	1.95	0.5
High BUN	0.87	0.77	0.65	1.7	0.55

Analysis of findings on erectile dysfunction

Severe ED was most frequent among individuals aged 42±12 years. While BMI and systolic blood pressure were similar across groups, diastolic pressure was greatest in severe ED (84±10 mmHg). Metabolic markers such as HbA1c and lipid profiles were identical, but severe ED cases showed mild TG increase (5.0±2.2 mmol/L). There was no significant difference in renal function markers except eGFR levels, which were mildly lower in severe ED (87±21 mL/min/1.73 m2). Severe ED patients had lower haemoglobin levels (11.2±2 g/dL) (Table 4).

**Table 4:** Erectile dysfunction and associated factors in diabetic patients (Mean ± SD) Statistical using test is t-test, HBA1c (haemoglobin A1c), Hb (haemoglobin), U.ACR (urinary albumin-to-creatinine ratio), TG (triglycerides), LDL (low-density lipoprotein), HDL (high-density lipoprotein), eGFR (estimated glomerular filtration rate), and BUN (blood urea nitrogen).

	Erectile dysfunction				p-value
	No erectile dysfunction	Mild erectile dysfunction	Moderate erectile dysfunction	Severe erectile dysfunction	
Age (years)	45±11	43±10	42±11	42±12	0.706
Height (cm)	160±10	159±11	159±16	160±7	0.821
Weight (Kg)	77±15	76±17	80±20	77±18	0.639
BMI (kg/m2)	30±7	31±7	32±10	30±7	0.865
Systolic blood pressure (mmHg)	126±10	125±10	120±10	125±9	0.461
Diastolic blood pressure (mmHg)	76±13	79±14	81±13	84±10	0.305
HBA1c	6±1	6±1	6±1	6±1	0.693
Fasting plasma glucose (mg/dL)	96±15	98±17	96±15	100±13	0.898
Hb level (g/dL)	12±2	12±2	13±2	11±2	0.483
Total cholesterol (mmol/L)	6±2	6±2	5±2	6±2	0.307
TG (mmol/L)	4.1±1.7	4.3±1.9	4.4±2.2	5.0±2.2	0.672
LDL (mmol/L)	3.0±2.2	3.7±2.1	4.1±2.1	2.7±1.6	0.257
HDL (mmol/L)	1.4±0.9	1.3±0.7	1.4±0.7	1.2±1.0	0.633
Serum creatinine (μmol/L)	76±18	77±24	89±23	85±25	0.306
eGFR (mL/min/1.73 m^2^)	95±21	92±17	95±16	87±21	0.686
U.ACR (mg/L)	50±36	66±35	68±38	55±34	0.245
BUN (mmol/L)	7±2	6±3	7±3	7±2	0.265
Blood uric acid (μmol/L)	272±72	273±66	257±69	266±74	0.097

**Figure 4:**
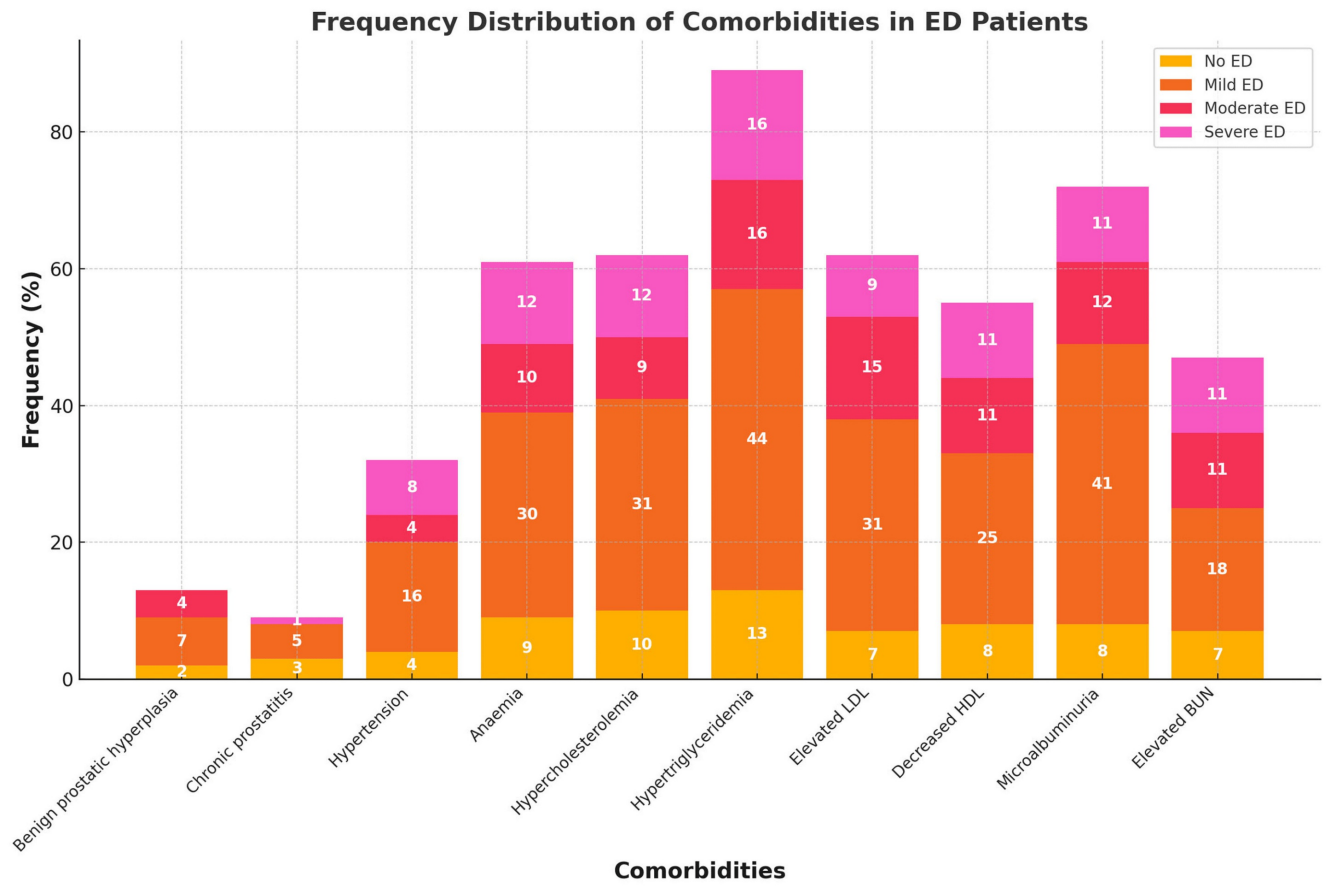
Frequency distribution of comorbidities in ED patients ED (erectile dysfunction), LDL (low-density lipoprotein), HDL (high-density lipoprotein), and BUN (blood urea nitrogen).

The most common form of ED was mild (49%), then moderate and severe ED (18% each). Married individuals made up most patients (42% with mild ED and 149% with severe ED). Obesity was among the most common factors, notably in mild (24%) and severe ED (9%). Hypertriglyceridemia (44%), hypercholesterolaemia (31%), and decreased HDL levels (25%) were prevalent for all ED severity. Microalbuminuria and anaemia were also prevalent in mild (41%) and severe ED (12%); however, moderate ED showed less involvement. Smoking showed a weaker link, as most ED patients were non-smokers (37%) (Table 5, Figure 4).

**Table 5:** Prevalence of demographics and lifestyle in erectile dysfunction (frequency and percentage of total) *Statistically significant by using Chi-Square test, BPH (benign prostatatic hyperplasia), Hb (haemoglobin), TG (triglycerides), LDL (low-density lipoprotein), HDL (high-density lipoprotein), eGFR (estimated glomerular filtration rate), DBP (diastolic blood pressure), CKD (chronic kidney disease), and BUN (blood urea nitrogen).

		Erectile dysfunction				p-value
		No erectile dysfunction	Mild erectile dysfunction	Moderate erectile dysfunction	Severe erectile dysfunction	
Age group	20-44 years	7 (7%)	24 (245)	10 (10%)	9 (9%)	0.958
	45-64 years	8 (8%)	25 (25%)	8 (8%)	9 (9%)	
Marital status	Married	13 (13%)	42 (42%)	13 (13%)	14 (14%)	0.560
Smoking status	Non-smoker	12 (12%)	37 (37%)	11 (11%)	14 (14%)	0.334
	Smoker	3 (3%)	12 (12%)	7 (7%)	4 (4%)	
BMI category	Underweight	0 (0%)	1 (1%)	1 (1%)	0 (0%)	0.930
	Normal weight	4 (4%)	13 (13%)	2 (2%)	5 (5%)	
	Overweight	4 (4%)	11 (11%)	5 (5%)	4 (4%)	
	Obese	7 (7%)	24 (24%)	10 (10%)	9 (9%)	
Type of diabetes	Type 1 diabetes	3 (3%)	9 (9%)	3 (3%)	1 (1%)	0.604
	Type 2 diabetes	12 (12%)	40 (40%)	15 (15%)	17 (17%)	
Duration of diabetic illness	Less than 10 years	6 (6%)	12 (12%)	5 (5%)	4 (4%)	0.03*
	More than 10 years	9 (9%)	37 (37%)	13 (13%)	14 (14%)	
Comorbidity	BPH	2 (2%)	7 (7%)	4 (4%)	0 (0%)	0.181
	Chronic prostatitis	3 (3%)	5 (5%)	0 (0%)	1 (1%)	0.083
	Hypertension (DBP≥ 90 mmHg)	4 (4%)	16 (16%)	4 (4%)	8 (8%)	0.517
	Anaemia (Hb < 13 g/dL)	9 (9%)	30 (30%)	10 (10%)	12 (12%)	0.924
	Hypercholesterolemia (˃ 5.2 mmol/L)	10 (10%)	31 (31%)	9 (9%)	12 (12%)	0.697
	Hypertriglyceridemia (TG˃ 1.7 mmol/L)	13 (13%)	44 (44%)	16 (16%)	16 (16%)	0.990
	Elevated LDL (≥2.6 mmol/L)	7 (7%)	31 (31%)	15 (15%)	9 (9%)	0.106
	Decreased HDL (<1.6 mmol/L)	8 (8%)	25 (25%)	11 (11%)	11 (11%)	0.832
	Microalbuminuria	8 (8%)	41 (41%)	12 (12%)	11 (11%)	0.065
	Elevated BUN	7 (7%)	18 (18%)	11 (11%)	11 (11%)	0.175
Creatinine (65-115 µmol/L)	Normal	10 (10%)	26 (26%)	12 (12%)	10 (10%)	0.524
	Elevated	0 (0%)	5 (5%)	3 (3%)	3 (3%)	
	Decreased	5 (5%)	18(18%)	3 (3%)	5 (5%)	
eGFR	G1 (normal, no CKD)	9 (9%)	25 (25%)	11 (11%)	7 (7%)	0.513
	G2 (No CKD)	6 (6%)	24 (24%)	7 (7%)	11 (11%)	
Total		15 (15%)	49 (49%)	18 (18%)	18 (18%)	

**Figure 5:**
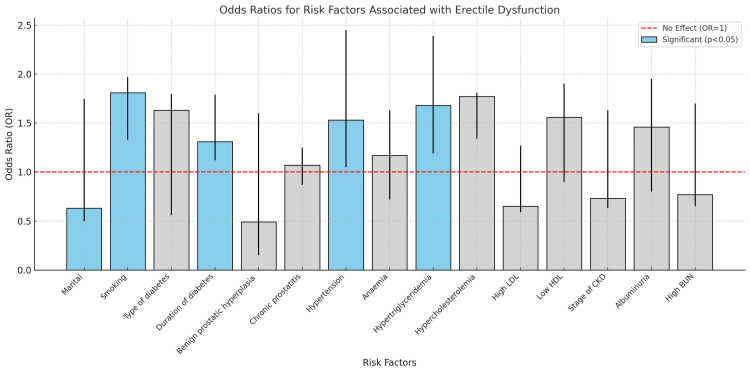
Odds ratio for risk factors associated with erectile dysfunction OD (odds ratio), RR (relative risk), CI (confidence interval), LDL (low-density lipoprotein), HDL (high-density lipoprotein), CKD (chronic kidney disease), and BUN (blood urea nitrogen).

ED was significantly associated with multiple risk factors. Marriage, smoking, hypertension, and long-term diabetes exhibited significant associations with ORs of 1.51, 1.48, 2.60, and 2.90, respectively. Furthermore, erectile dysfunction was significantly associated with elevated cholesterol (OR = 2.50), increased LDL (OR = 1.80), and decreased HDL (OR = 1.70). ED was not significantly predicted by BPH (OR = 0.97), chronic prostatitis (OR = 0.80), or albuminuria (OR = 0.38) (Table 6, Figure 5).

**Table 6:** Odds ratio for risk factors associated with erectile dysfunction The data was statistically significant using logistic regression, OR (odds ratio), RR (relative risk), CI (confidence interval), LDL (low-density lipoprotein), HDL (high-density lipoprotein), CKD (chronic kidney disease), and BUN (blood urea nitrogen).

Risk Factor	RR	OR	95% CI Lower	95% CI Upper	p-value
Marital	1.66	1.51	1.20	2.05	0.04*
Smoking	1.49	1.48	1.10	1.90	0.04*
Type of diabetes	1.50	1.39	0.64	1.89	0.70
Duration of diabetes	3.00	2.90	1.50	3.50	0.02*
Benign prostatic hyperplasia	0.97	0.97	0.49	1.87	1.00
Chronic prostatitis	0.40	0.30	0.17	0.84	0.13
Hypertension	2.70	2.60	1.50	2.60	0.77
Premature Ejaculation	2.20	2.09	1.45	2.60	0.04*
Anaemia	0.96	0.95	0.43	1.92	1.00
Hypertriglyceridemia	2.55	2.50	2.15	2.97	0.03*
Hypercholesterolemia	2.30	2.27	1.90	2.64	0.04*
High LDL	1.87	1.80	1.30	2.90	0.02*
Low HDL	1.78	1.70	1.40	2.80	0.01*
Stage of CKD	1.44	1.40	0.86	1.87	0.54
Albuminuria	0.44	0.38	0.12	1.16	0.12
High BUN	0.99	0.98	0.33	1.36	1.00

## Discussion

The prevalence of ED among male diabetic patients in Saudi Arabia is notably high. 85% of our patients had ED (49% had mild ED, 18% moderate ED, and 18% severe ED). Data from King Khalid University Hospital in Riyadh, which reported 75.2% of diabetic men with ED - 64% partial and 11.2% severe - aligns with these findings [18]. Another Saudi Arabian study found that diabetic men with low testosterone levels had an even higher ED prevalence of 86.7% [19]. A multicenter study in Jeddah revealed that 30% of patients with ED had diabetes [18]. Mild to moderate cases of ED in our study had borderline increases in diastolic blood pressure (84±10 mmHg) and low eGFR values (87±21 mL/min/1.73 m2), which may suggest some vascular and renal changes. Moreover, TG levels had higher values in severe cases of ED (5.0±2.2 mmol/L), suggesting that ED may have a continuum with an apparent role of lipid metabolism in propagation. The level of haemoglobin was the lowest in the severe cases of ED (11±2 g/d/L), indicating that there is a possibility that anaemia is a cause.

Forty-seven percent of our patients had PE, and 7% had borderline PE. The mean ejaculatory latency time was shorter in diabetic patients (3.6±2.7 minutes) compared to non-diabetic individuals (4.3±2.8 minutes) [8]. PE is found to be significantly more prevalent in diabetic patients compared to non-diabetics [8]. Although the search results don't offer precise information about the prevalence of PE in Saudi patients with diabetes, one study reported that PE was present in 75.8% of male Saudi diabetic patients, and another study reported the prevalence to be 83% [8, 20].

We noted the co-occurrence of ED and PE in 39% of our cases (OD = 2.09, CI = 1.45-2.60, p<0.05). A study of diabetic patients in Saudi Arabia found that those with ED showed a significantly higher incidence of PE and shorter ejaculatory latency times [8].

Sixteen percent of our studied patients had type 1 diabetes, while 84% had type 2 diabetes. Both type 1 and type 2 diabetes are associated with an increased risk of sexual dysfunction in men, particularly ED. We observed ED in 49% of patients with type 1 diabetes and 86% of patients with type 2 diabetes. Research indicates that 45.8% of men with both types of diabetes experience ED, which is nearly double the rate in men without diabetes (24.1%) [21]. Some studies have shown that 61.8% of men with type 1 diabetes report poor erectile function [21]. Additionally, other studies suggest that men with type 1 diabetes may have a higher risk of developing ED compared to those with type 2 diabetes [9]. 

We found that 37% of patients with type 1 diabetes and 81% of patients with type 2 diabetes had PE. Despite not being specific to Saudi Arabia, a study of type 2 male diabetic patients found the prevalence of PE to be 27.5% [22], while another study reported it to be 40.2% [23].

In this study, the results confirm the presence of a strong relationship between some risk factors and the prevalence of ED and PE in the population under review. More specifically, the presence of one or more of these three risk factors was found to predispose ED: marital status, smoking, and duration of diabetes, with ORs of 1.51, 1.48, and 2.90, respectively, all statistically significant at p < 0.05. This indicates that these factors are important contributors to the risk of developing ED. Similarly, smoking, marital contact, and long-standing diabetes were also associated with the occurrence of PE with ORs of 0.63 (p = 0.04), 1.81 (p = 0.04), and 1.31 (p = 0.01), respectively. The higher incidence of such sexual dysfunction among married patients reflects potential psychosocial stressors. While marriage itself is not explicitly identified as a risk factor for sexual dysfunction in diabetic men, the quality of the marital relationship and associated psychosocial factors can significantly influence sexual health outcomes [24]. Sexual function is significantly affected by diabetes duration, particularly when it exceeds 10 years. After more than 10 years of diabetes, 38% of our patients had PE and 64% had ED. After 10 years of having type 2 diabetes, the risk of ED becomes significantly greater compared to men without diabetes [21]. Men with diabetes lasting more than 10 years were 2.7 times more likely to report PE compared to those with diabetes for less than 5 years [23]. 

Also, other conditions such as hypertension (OR = 2.60, p< 0.05) were reported to have a strong relationship with ED, which underscores the need to control cardiovascular comorbidities in order to reduce sexual dysfunction.

According to our study, the proportion of patients with elevated triglycerides was 76% among those with ED and 43% among those with PE, with odds ratios of 2.5 and 1.68, respectively, indicating a significant relationship (p < 0.05). Furthermore, our studies revealed that there were also important relationships between ED and hypercholesterolaemia (OR = 2.50, p < 0.05), elevated LDL cholesterol levels (OR = 1.80, p < 0.05), and low HDL cholesterol levels (OR = 1.70, p < 0.05). Diabetic patients frequently have a specific lipid abnormality known as diabetic dyslipidaemia, which is characterised by elevated levels of TG, decreased levels of HDL, and elevated levels of LDL [25]. This dyslipidaemia is associated with an increased risk of future cardiovascular disease and an increase in the severity of one’s sexual dysfunction [25]. Studies have shown that hyperlipidaemia affects the smooth muscle and endothelium cells within the penis, resulting in impaired erectile function [26]. Besides, increased levels of LDL have been associated with arteriogenic ED. Research has shown that 2.41 mmol/L of LDL levels is a determinant in over 55.2% and up to 76.7% of cases of arteriogenic ED [27]. Lastly, the presence of HDL at low levels among diabetic patients may precipitate ED. Higher HDL levels may help protect against arteriogenic ED because there is a positive relationship between HDL levels and peak systolic velocity in penile arteries [27].

On the contrary, in our study, BPH (OR = 0.97, p = 1.00), chronic prostatitis (OR = 0.80, p > 0.05), and albuminuria (OR = 0.38, p > 0.05) were found to have weak associations with ED. In addition, these variables - BPH (OR = 0.49, p = 0.25), chronic prostatitis (OR = 1.07, p = 1.00), and albuminuria (OR = 1.46, p = 0.50) - were also shown to be statistically insignificant in predicting PE. Based on these findings, it can be inferred that these variables are not important in predicting either ED or PE attacks.

Adding to this, renal markers such as serum creatinine, eGFR, and urinary albumin-to-creatinine ratios were consistent throughout the groups, excluding the role of significant renal dysfunction in the prevalence of PE or ED. However, borderline PE patients showed slightly lower haemoglobin levels (11±3 g/dL), suggesting anaemia could be a mildly influential factor.

Limitations

The cross-sectional design limits causal inference. Additionally, the sample size of 100 participants restricts the generalisability of the findings. Future studies with larger, longitudinal designs are needed to confirm these associations and explore underlying mechanisms.

## Conclusions

The increased incidences of ED and PE emphasise the importance of a proper diagnostic approach. An important step would be the approaches to quitting smoking, controlling blood sugar levels, managing lifestyle habits, and regulating lipids. Screening for sexual dysfunctions and assessment of lipid profiles should be part of the diabetes management in order to enhance the life of the patient.
